# A framework for artificial intelligence in cancer research and precision oncology

**DOI:** 10.1038/s41698-023-00383-y

**Published:** 2023-05-17

**Authors:** Raquel Perez-Lopez, Jorge S. Reis-Filho, Jakob Nikolas Kather

**Affiliations:** 1 Radiomics Group, Vall d’Hebron Institute of Oncology, Vall d’Hebron Barcelona Hospital Campus, Barcelona, Spain; 2 Department of Pathology, Memorial Sloan Kettering Cancer Center, New York, NY USA; 3 Else Kroener Fresenius Center for Digital Health, Technical University Dresden, Dresden, Germany; 4 Department of Medicine I, University Hospital Dresden, Dresden, Germany; 5 Medical Oncology, National Center for Tumor Diseases (NCT), University Hospital Heidelberg, Heidelberg, Germany

## Introduction

This collection aims to disseminate the latest research and advancements in all aspects of artificial intelligence (AI) within the realm of cancer research, including basic, translational, and clinical research. The collection is undoubtedly ambitious, as it seeks to present a comprehensive review of current AI applications in precision oncology while offering expert insights on accelerating the transition of AI tools from the laboratory to the clinic, with the aim of ultimately enhancing patient care and improving clinical endpoints. Priority will be given to articles that employ innovative methodologies, address pertinent real-world issues, and provide robust evidence through the utilization of multicentric datasets. Although the definition of AI remains ambiguous, most contemporary, successful AI systems incorporate some form of deep learning^1^. Within the biomedical research literature, there is a prevailing consensus that the term “AI” can be applied to deep learning approaches—those involving models with millions or billions of parameters—rather than simple statistical models such as linear regression with only a few free parameters and co-variables^1^. Although deep learning approaches are the primary focus of this collection, we remain receptive to related fields.

## Image processing

Medicine and oncology, in particular, are abundant with image data^2,3^. For example, skin tumors are diagnosed visually and can be identified from digital photographs. Numerous cancer types, such as those of the digestive tract, are diagnosed endoscopically. Breast cancer, and many other tumor types, are screened for and detected by radiology imaging. The definitive diagnosis of cancer is made through a process of histopathology based on the analysis of images from stained tissue. Radiology images are also commonly used in the staging and evaluation of responses to cancer treatments. In essence, the heavy reliance of oncology on image analysis makes the acquisition, interpretation, and analysis of such images critical at all stages of the cancer detection, characterization, and follow-up processes. Over the last century, a critical revolution has taken place in the field of medical imaging processing and storage, transitioning from analog detectors in radiology to digital detectors. Additionally, there has been an extensive expansion in the efforts of pathology departments in the digitalization of tumor samples. This shift toward digitalization has opened up new and exciting opportunities for the storage, sharing, and use of image data. With digital image data, powerful computational AI methods can be applied, enabling high-throughput analysis and exploitation of data that was once only subjectively assessed by medical experts. As a result, the digitization of medical imaging represents a major breakthrough and promises significant advances in oncology. Prior to 2012, computer-based image analysis was a formidable challenge, and computer-based methods were highly restricted to very narrow domains and datasets. With the breakthrough of AlexNet^4^ as the first prominent convolutional neural network (CNN) in 2012, deep learning has since become the method of choice for virtually any image analysis task. Consequently, in oncology, the vast majority of AI solutions are related to the analysis of digital images. AI can automate human tasks, such as cancer detection on endoscopy videos, radiology images, or histopathology slides. Numerous programs have received regulatory approval in Europe, the US, and other markets and can now be utilized in patients’ routine care. AI can, however, transcend these applications by yielding image-based biomarkers capable of directly predicting survival or treatment response, thus extracting considerably more information from image data than was previously possible^5,6^. Such biomarkers could be integrated into prognostic or predictive models that are already utilized in clinical care, thus acting as integrative biomarkers. In this collection, we aim to explore all these approaches spanning cancer diagnosis, treatment, and follow-up.

## Language processing

The text serves as the universal interface for communication among humans and between humans and machines. Electronic health records, patient-reported outcomes, and communication between healthcare providers and patients all rely on text. In oncology, this implies that a significant portion of diagnostic and clinical workflows generates text artifacts^7^. Physicians often devote a considerable amount of time to reading text documents describing patients’ previous medical histories or diagnostic reports. Until recently, this vast resource remained largely untapped by digital tools. The field of natural language processing (NLP) has developed numerous methods for computer-based text mining, but none were particularly robust, effective, and generalizable. Tasks such as summarizing multi-page medical reports, translating languages, or converting writing styles were considered highly complex. With the advent of transformer neural networks, initiated by the groundbreaking paper “Attention is All You Need“^8^, exponential growth in NLP has been spurred. By employing transformers with tens or hundreds of billions of parameters (known as large language models, or LLMs) and training them on vast amounts of data (primarily all available internet text), human expert-level performance can be achieved for nearly any language understanding or creation task. In an unexpected twist of history, LLMs have also inadvertently demonstrated remarkable logical reasoning skills^9^. The transformative impact of this technology on all facets of society, including cancer research and oncology, cannot be overstated. The introduction of LLMs will likely lead to an exponential increase in overall productivity and pace in the field, much like the introduction of the Internet. This does not imply that cancer researchers or oncologists will be rendered obsolete; rather, the demand for human orchestration in the process will continue. In this collection, we encourage the submission of scientifically rigorous articles that employ LLM-based methods in oncology and provide empirical data supporting their benefits for research and patient care.

## Enhancing genomic information with AI

The use of next-generation sequencing (NGS) has revolutionized cancer research and drug development in oncology, leading to unparalleled precision and success. AI tools open tremendous possibilities to leverage high-throughput genomics data, including whole-genome, whole-exome, or whole-transcriptome analysis, with the promise not only to enhance the clinical practice but also to acquire valuable insights into cancer biology^10^. The implementation of AI-based techniques for the analysis of genomics data can identify novel genomic and transcriptomic features with prognostic and predictive significance in cancer, as well as enable large-scale analysis of pathogenicity of variants and genotype–phenotype associations. Importantly, AI applications in cancer genomics could unlock a new level of decision-support tools enabling truly personalized care. There remain several challenges that need to be addressed in the adoption of AI analytics in genomics. With this in mind, we encourage the submission of articles detailing clinically meaningful discoveries in genomics using AI, as well as new methods for tackling encountered challenges.

## The future is multimodal

The tremendous success of transformer neural networks has brought another once-elusive goal within reach: multimodal machine learning models^11^. These models can accept different types of data as a simultaneous input, such as various image types, images combined with text, or images integrated with genomic data. Intuitively, it is clear that oncological decision-making is inherently multimodal. Oncologists do not prescribe treatments based solely on a single image; rather, they consider multiple pieces of information and corresponding data. Combining different data types can yield more than just the sum of their parts. Not uncommonly, a pattern in an image only becomes meaningful in light of the genetic make-up of the patient, a given cancer genomic alteration, and/or medical history. Training machine learning models on multimodal data was once considered too ambitious. This changed recently, as several publications in 2022 and early 2023 demonstrated sensible applications of multimodal models in oncology^12,13^. By utilizing transformer neural networks, these approaches have become more powerful and versatile^14^. While such approaches present regulatory and practical challenges, we believe that their implementation is imminent. In this collection, we encourage the submission of articles exploring novel technical approaches for multimodality in relevant oncological applications.

## The primacy of evidence-based medicine and patient-centered care

This collection aims to provide a comprehensive review of current AI applications in precision oncology and explore novel technical approaches. The overarching aim is the use of AI to generate solid scientific evidence which can improve cancer research and patient care. We currently live in an era of unprecedented technological progress, with no signs of slowing down^15^, and this collection aims to reflect these developments. However, we emphasize that the practice of medicine transcends technological advancement; at its core, it involves empathetic human interactions, shared decision-making based on rigorous science, and consideration of patient preferences. These principles must guide any research we undertake, making it imperative that AI in oncology respects and advances the primacy of evidence-based medicine and patient-centered care. We must be mindful of these principles, and in addition, we must include ethical considerations, data privacy concerns, and potential biases in the development, evaluation, and clinical implementation of AI systems.

## Methods

In accordance with the COPE (Committee on Publication Ethics) position statement of 13 February 2023 (https://publicationethics.org/cope-position-statements/ai-author), the authors hereby disclose the use of the following artificial intelligence models during the writing of this article. GPT-4 (OpenAI) for checking spelling and grammar.
